# 

**Published:** 1913-06

**Authors:** 


					CONTENTS. Page i|
On the Course of Pernicious Anaemia in Older Persons.
By T.
Michell Clarke, M.A., M.D., LL.D., F.R.C.P.
97
Methods of Diagnosis in Gastric Cancer.
By J. M. Fortescue-
Brickdale, M.A., M.D. Oxon.
io8
Paroxysmal CEdema of the Lungs.
By C. E. S. Flemming,
M.R.C.S. Eng., L.R.C.P. Lond.
Notes on two Cases of Urinary Calculus.
By C. A. Moore,
M.S.Lond., Ch.M. Bristol., F.R.C.S.
126
Urinary Products of Intestinal Intoxication.
By G. Hely-Hotchinson
Almond, M.A., B.M., B.Ch. Oxon. ..
13?
Some Recent Cases of Epidemic Cerebro-Spinal Meningitis.
J. R. Charles, M.A., M.D.Cantab., F.R.C.P. ...
142
By
?n the Occurrence of Serous Pleurisy due to Pneumococci and
Tubercle Bacilli.
By F. H. Edgeworth, M.D.Cantab., D.Sc. Lond.
146
What is an Accident ?
By Charles Corfield, of the Middle Temple,
Barrister-at-Law, M.R.C.S., L.R.C.P.
148
*ne Tuberculosis Officer and his Work.
By Latimer J. Short,
M.D., B.S.Lond., M.D.Bristol.
153
'he Third South Midland Field Ambulance.
By Lieut.-Colonel
A. W. Prichard, V.D.
159
Progress of the Medical Sciences:
Surgery.
By James Swain, M.S., M.D.Lond.
165
Pathology.
By G. Scott Williamson, L.R.C.P., L.R.C.S. Edin.
i68
Reviews of Books
172
Notes on Preparations for the Sick
i8o
Library of the Bristol Medico-Chirurgical Society
183
Meetings of Societies
i86
Obituary Notice
189
l-ocal Medical Notes
192

				

